# Ureteral involvement in an inguinal hernia: the importance of preoperative identification

**DOI:** 10.1093/jscr/rjag977

**Published:** 2026-10-27

**Authors:** Mohammad N Huda, Ponnuthurai Pratheepan, Michael F Bath, Abdulrahman Mohamed, Mohammed Obaid, Tun Tha, Lee Dvorkin, Harshawardhan Godbole

**Affiliations:** Department of Urology, North Middlesex University Hospital, Sterling Way, London, N18 1QX, United Kingdom; Department of Surgery, North Middlesex University Hospital, Sterling Way, London, N18 1QX, United Kingdom; Department of Surgery, North Middlesex University Hospital, Sterling Way, London, N18 1QX, United Kingdom; Department of Engineering, University of Cambridge, Cambridge, CB2 1PZ, United Kingdom; Department of Radiology, North Middlesex University Hospital, Sterling Way, London, N18 1QX, United Kingdom; Department of Radiology, North Middlesex University Hospital, Sterling Way, London, N18 1QX, United Kingdom; Department of Radiology, North Middlesex University Hospital, Sterling Way, London, N18 1QX, United Kingdom; Department of Surgery, North Middlesex University Hospital, Sterling Way, London, N18 1QX, United Kingdom; St George’s University School of Medicine, True Blue Campus, Grenada, West Indies; Department of Urology, North Middlesex University Hospital, Sterling Way, London, N18 1QX, United Kingdom; St George’s University School of Medicine, True Blue Campus, Grenada, West Indies

**Keywords:** hernia surgery, urology, ureter

## Abstract

A ureteroinguinal hernia is a rare clinical finding, particularly in those without any previous abdominal surgery. We report the case of an 80-year-old male, with no previous abdominal surgery, who presented with a large symptomatic right-sided inguinoscrotal hernia, associated with lower urinary tract symptoms. Preoperative computed tomography demonstrated a mild-to-moderate right-sided hydronephrosis, with the distal ureter tapering and looping into the inguinal hernia. Intraoperative retrograde pyelography confirmed a characteristic ‘fish hook’ configuration of the distal ureter. The patient underwent an elective open right inguinal hernia repair with a preprocedural intraoperative ureteric stent placement to ensure sufficient identification, preservation, and reduction of the ureter during the operation. Postoperative imaging demonstrated sufficient straightening of the ureter and the patient had an uneventful recovery. This case highlights the importance of preoperative imaging and multidisciplinary planning in patients with inguinal hernia and ipsilateral hydronephrosis in order to prevent any inadvertent ureteric injury.

## Introduction

Over 100 000 hernia repairs are performed annually in the UK [1], and inguinal hernia repair is one of the most common surgical procedures performed globally [2]. Inguinal hernia will typically contain intra-abdominal fat or bowel, however a variety of intra-abdominal viscera has previously been described—involvement of a ureter within an inguinal hernia is very uncommon.

A select number of cases describing ureteric involvement in an inguinal hernia have previously been reported, though predominantly in patients with a history of kidney transplantation, given the anterior location of the transplanted ureter [3]. However, such cases are much less common in those who have not had previous surgery. Ureteral involvement appears to be more common on the right side, presumed to be due to the fascia of Toldt on the left side at the level of the sigmoid mesocolon root, tightening and fixing the ureter in the retroperitoneum.

We present the case of a patient presenting with a large right-sided inguinoscrotal hernia containing the ureter, who had not previously had any abdominal surgery. Having been identified preoperatively, the patient was therefore able to undergo appropriate preoperative and intraoperative preparations to ensure a safe hernia surgery could be performed.

## Case report

An 80 year old male was referred to the outpatient general surgery clinic by the urology team due to a symptomatic right-sided inguinoscrotal swelling. At this time, he further reported associated urinary frequency, urgency, and nocturia. He had a past medical history of atrial fibrillation, heart failure, and chronic kidney disease (stage 3), with no previous abdominal surgery. On examination, a large irreducible right inguinoscrotal hernia was present, with the rest of the abdomen soft and non-tender.

A preoperative computed tomography (CT) scan of the chest abdomen and pelvis with intravenous contrast and subsequent noncontrast CT kidneys, ureters, and bladder scan were performed as part of the preoperative work-up (Fig. 1). A mild-to-moderate right side pelvicaliceal dilatation was noted, with the distal ureter tapering as it entered into a right-sided inguinal hernia, alongside some preperitoneal fat also present. A preoperative MAG3 scan was subsequently performed which, revealed a divided right renal function of 18.7% (Fig. 2).

**Figure 1:**
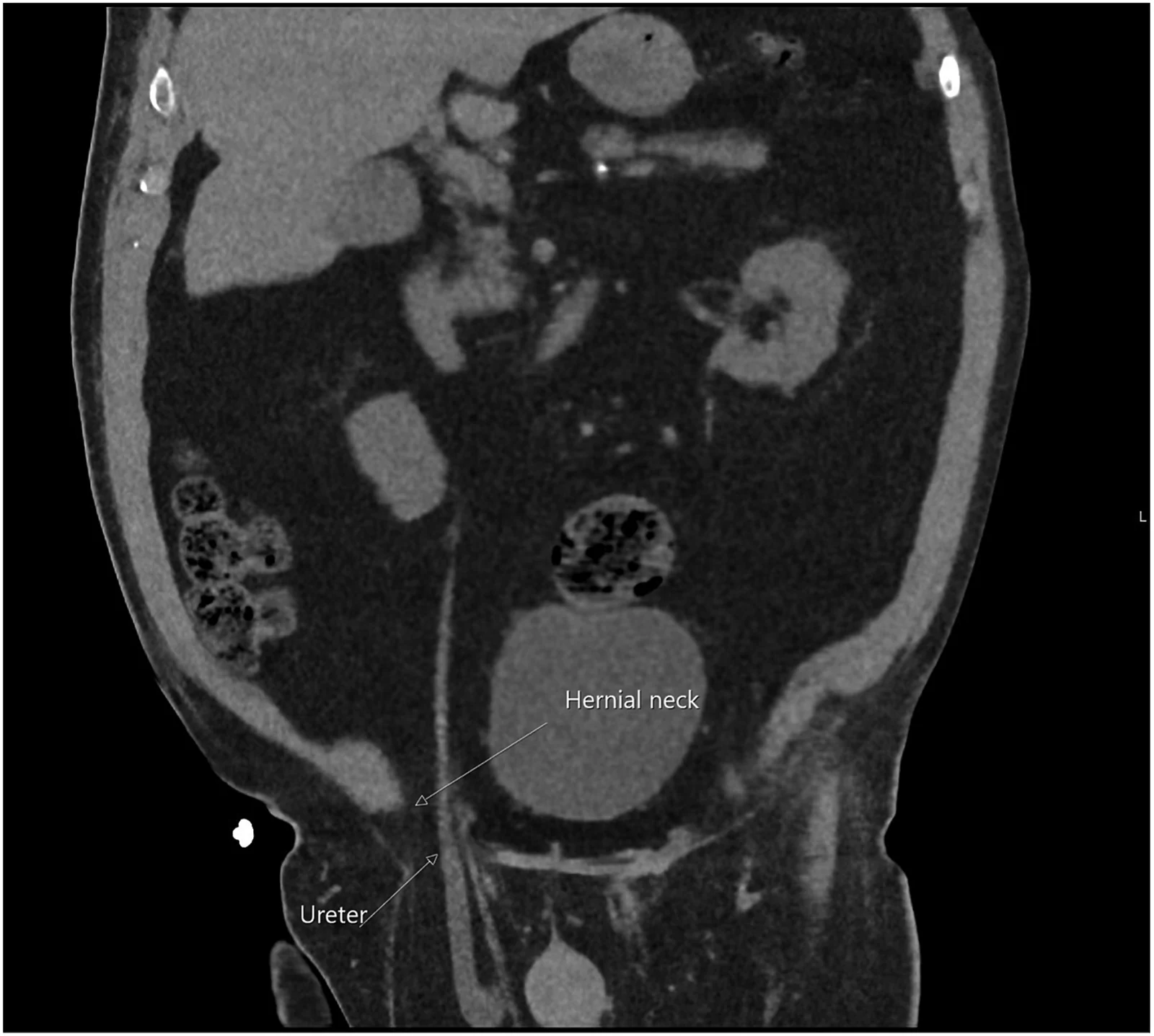
Pre-operative non-contrast CT scan, demonstrating the right ureter entering the right inguinal hernia.

**Figure 2:**
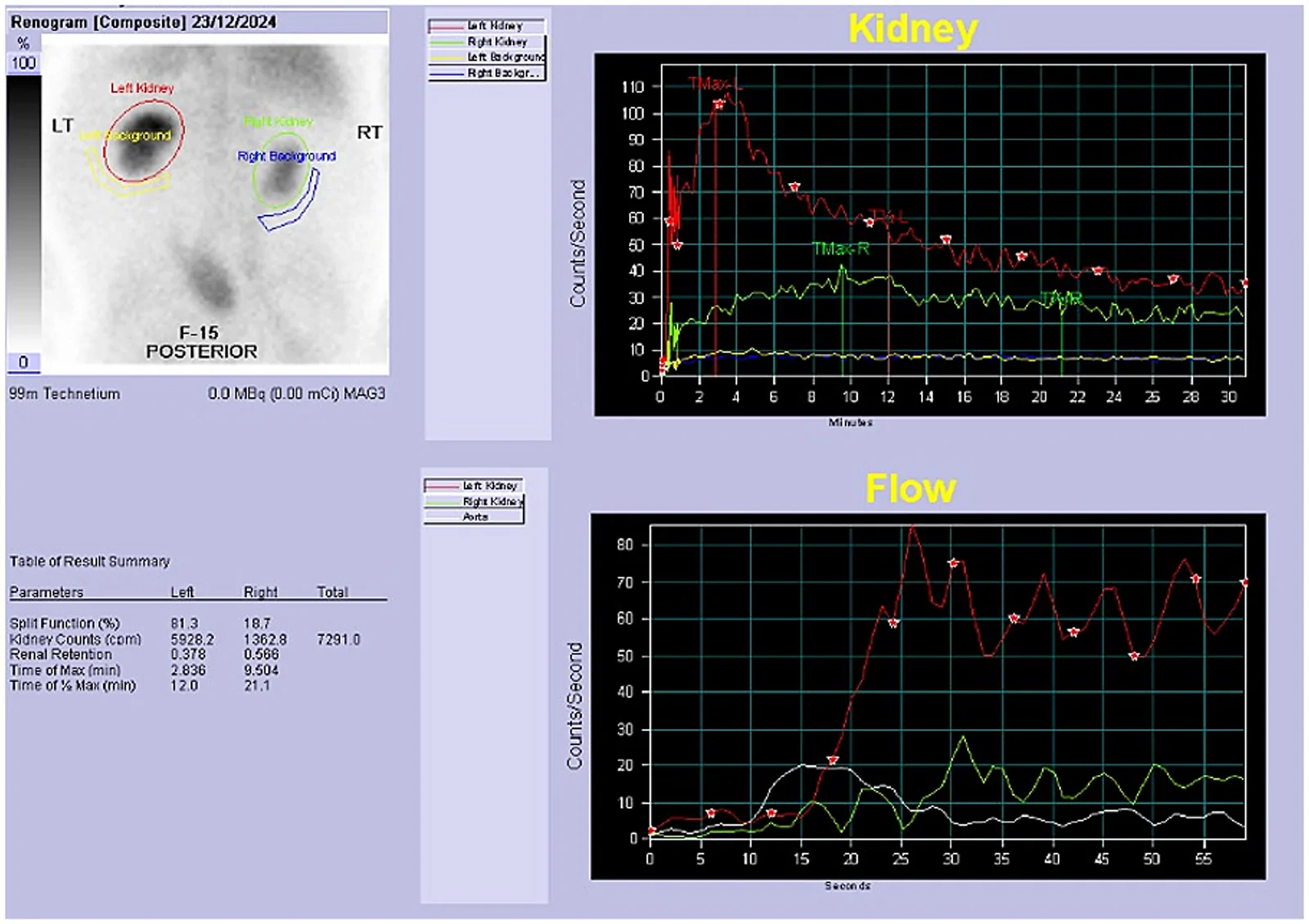
MAG-3 renogram demonstrating reduced split function contribution of the right kidney compared to the left.

The patient was listed for a routine open right groin hernia repair with mesh. Whilst under general anaesthesia but prior to the hernia repair, a rigid cystoscopy and right ureteric stent was successfully inserted; an intraoperative retrograde pyelogram was performed (Fig. 3), which noted a “fish hook” appearance of the distal ureter, with looping of the distal right ureter within the inguinal hernia.

**Figure 3:**
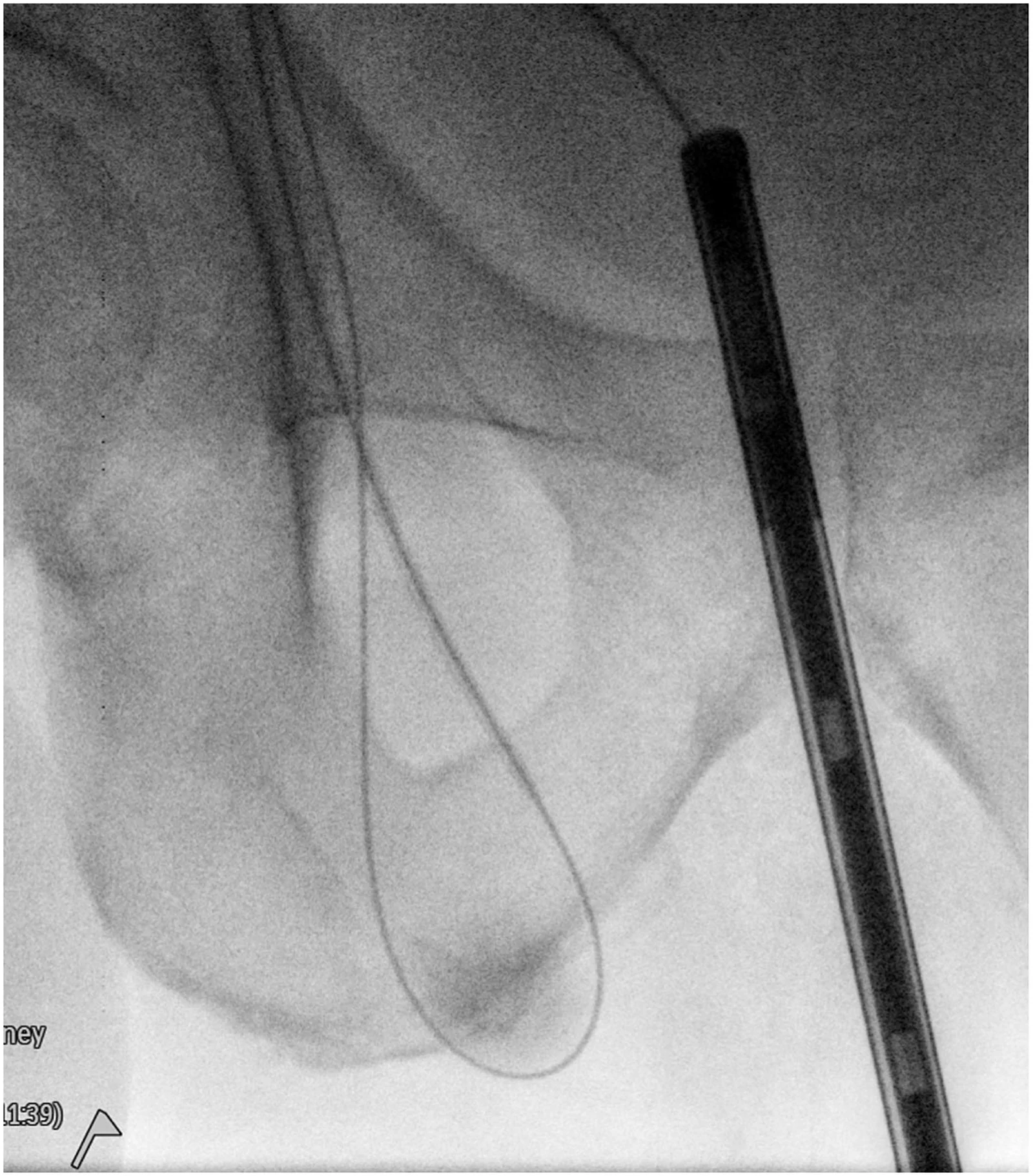
Retrograde pyelogram findings of ureter in inguinal hernia.

During the subsequent open hernia repair, a sliding direct hernia was identified. The vas deferens and vessels were identified, with the right ureter (containing its palpable stent in situ) able to be preserved throughout (Fig. 4). To ensure appropriate anatomical views were obtained during the hernia repair, a slightly larger skin incision had to be made and the testicle was brought out from the scrotum into the wound during the operation. The hernial component was separated and reduced (of both bowel and ureter) to allow the posterior inguinal wall to be plicated and a Lichtenstein mesh repair to then be performed.

**Figure 4:**
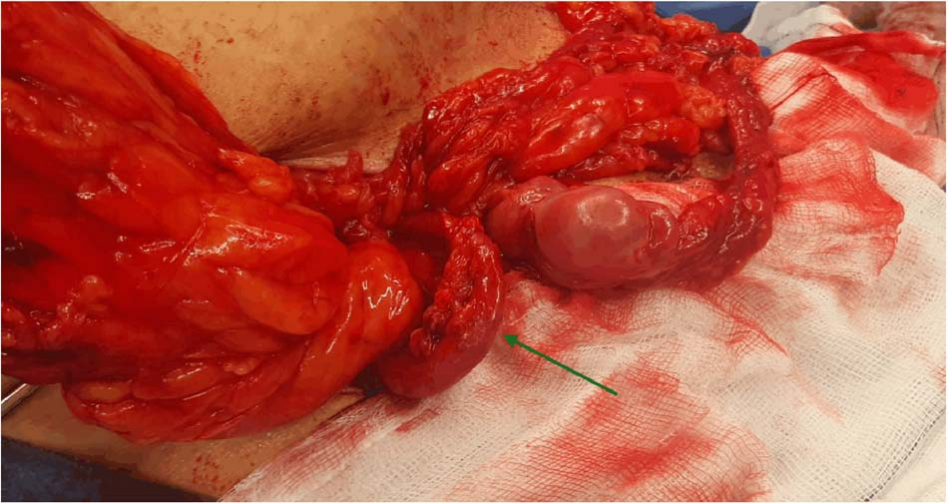
Intraoperative photo highlighting the position of the ureter (marked with arrow).

The patient was discharged and was reviewed in the outpatient clinic one month later, where his hernia wound was noted to have fully healed, with no signs of clinical recurrence. Blood tests performed just 2 months postoperatively demonstrated a modest increase of the eGFR (estimated glomerular filtration rate) to 44 ml/min, from a preoperative eGFR of 37 ml/min. A retrograde pyelogram performed 8 months postoperatively showed a straightened right ureter (Fig. 5), to allow for his ureteric stent to then be successfully removed.

**Figure 5:**
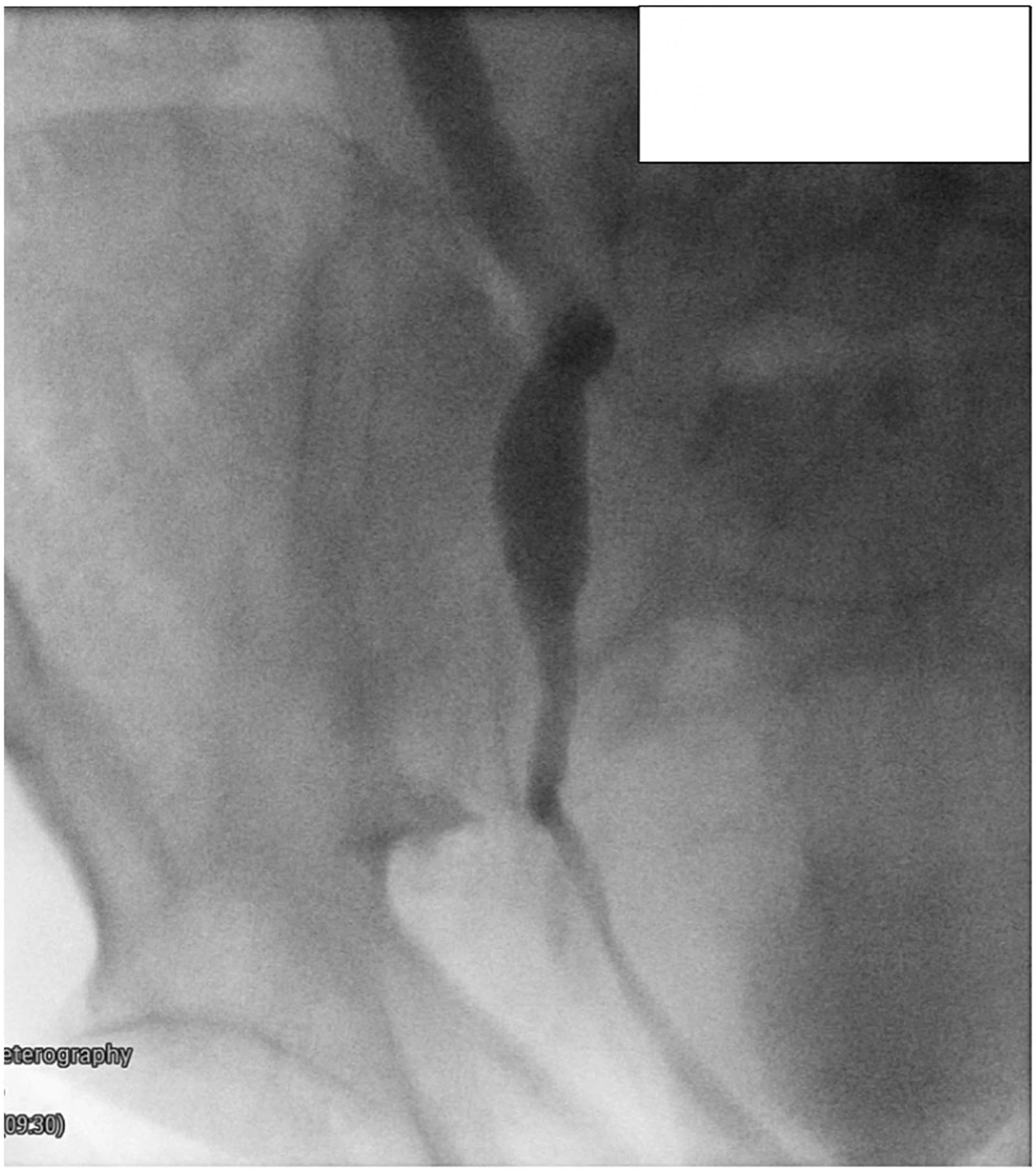
Retrograde pyelogram demonstrating the right ureter in the anatomical position.

## Discussion

In this case report, we describe the identification of a ureter in a right-sided inguino-scrotal hernia preoperatively and the subsequent interventions that were undertaken to ensure a successful repair. Following the repair, not only was there no evidence of clinical recurrence of the hernia, but there was a slight improvement in the patient's overall renal function. This patient had no previous history of abdominal surgery, however due to clinical diligence, the ureteric involvement in the hernia was identified preoperatively and through a collaborative approach between general surgeons and urologists, a successful outcome for the patient was achieved.

Ureteroinguinal hernias are rare in patients with native kidneys. For the limited number identified in the wider literature, they are generally divided into paraperitoneal and extraperitoneal variants [4, 5]. Paraperitoneal-type ureteroinguinal hernias, which was the case in this patient, occur due to adhesion between the ureter and the herniating peritoneal sac, whilst extraperitoneal-type hernias occur due to congenital fusion between the ureter and the genitoinguinal ligaments. Patients with previous hernia surgery are likely to be affected by such pathology, whereby scarring draws the ureter into the hernia sac. One unique aspect of this case report derives from the patient not having had any previous surgery before; previous theories have suggested that potentially the anterior displacement of the ipsilateral ureter from psoas muscle at L4 level can cause such a pathology to occur [6].

An inguinal hernia containing a ureter does not lead to strangulation and obstructive uropathy often, due to the large size of majority of these inguinal hernias and the sufficiently wide hernial necks that prevent any trapping of the ureter [6, 7]. The condition is instead often diagnosed incidentally, either during or after herniorrhaphy, when the ureter can be inadvertently injured. In the case described, ureteral involvement was successfully identified during preoperative investigation for hydronephrosis, meaning appropriate intraoperative intervention and precautions could take place. As such, an important point to emphasize, that in the presence of an ipsilateral hydronephrosis with concurrent inguinal hernia, the clinician should alert to the potential presence of ureter in the hernia.

Though a ureter within an inguinal hernia is a rare occurrence, the current case demonstrates the importance of preoperative evaluation for diagnosis to ensure inadvertent ureteric injury is avoided.
